# The empowerment of translational research: lessons from laminopathies

**DOI:** 10.1186/1750-1172-7-37

**Published:** 2012-06-12

**Authors:** Sara Benedetti, Pia Bernasconi, Enrico Bertini, Elena Biagini, Giuseppe Boriani, Cristina Capanni, Nicola Carboni, Giovanna Cenacchi, Marta Columbaro, Monica D'Adamo, Adele D’Amico, Maria Rosaria D’Apice, Marianna Fontana, Alessandra Gambineri, Giovanna Lattanzi, Rocco Liguori, Nadir M Maraldi, Laura Mazzanti, Eugenio Mercuri, Tiziana Mongini, Lucia O Morandi, Iria Neri, Giovanni Nigro, Giuseppe Novelli, Michela Ortolani, Renato Pasquali, Antonella Pini, Stefania Petrini, Luisa Politano, Stefano Previtali, Lisa Pucci, Claudio Rapezzi, Giulia Ricci, Carmelo Rodolico, Paolo Sbraccia, Emanuela Scarano, Gabriele Siciliano, Stefano Squarzoni, Antonio Toscano, Liliana Vercelli, Matteo Ziacchi

**Affiliations:** 1 Laboratory of Molecular Biology, Diagnosis and Research San Raffaele, San Raffaele Scientific Institute, Milan, Italy; 2 Neuroimmunology and Neuromuscular Disorders Unit, Foundation IRCCS Neurological Institute Carlo Besta, Milan, Italy; 3 Bambino Gesù Hospital, Rome, Italy; 4 Institute of Cardiology, University of Bologna and Azienda Ospedaliera S.Orsola Malpighi, Bologna, Italy; 5 National Research Council of Italy, Institute of Molecular Genetics, IGM-CNR, Unit of Bologna, via di Barbiano 1/10, 40136, Bologna, Italy; 6 Neuromuscular Unit, Department of Cardiological and Neurological Sciences, University of Cagliari, Cagliari, Italy; 7 Clinical Department of Radiological and Histopathological Sciences, University of Bologna, Bologna, Italy; 8 Laboratory of Musculoskeletal Cell Biology, Istituto Ortopedico Rizzoli, Bologna, Italy; 9 Department of Internal Medicine, University of Rome Tor Vergata, Rome, Italy; 10 Fondazione Policlinico Tor Vergata, Rome, Italy; 11 Fondazione G. Monasterio, CNR-Regione Toscana, Pisa, Italy; 12 Division of Endocrinology, S.Orsola-Malpighi Hospital, University of Bologna, Bologna, Italy; 13 IRCCS, Istituto delle Scienze Neurologiche di Bologna, Department of Neurological Sciences, Bologna University, Bologna, Italy; 14 Department of Paediatrics, University of Bologna, Bologna, Italy; 15 Department of Paediatrics, Child Neurology and Psychiatry, Catholic University, Rome, Italy; 16 Center for Neuromuscular Diseases, Department of Neuroscience, University of Turin, Turin, Italy; 17 Department of Internal Medicine, Aging and Nephrological Diseases, Dermatology, University of Bologna, Bologna, Italy; 18 Department of Experimental Medicine, Cardiomyology and Medical Genetics II, University of Naples, Naples, Italy; 19 San Pietro Fatebenefratelli Hospital, Rome, Italy; 20 UOC Paediatric Neuropsychiatry, Bellaria-Maggiore Hospital, Bologna, Italy; 21 Division of Neuroscience and Institute of Experimental Neurology (INSPE), San Raffaele Scientific Institute, Milan, Italy; 22 Diatheva SRL, Fano (PU), Italy; 23 Department of Neuroscience, University of Pisa, Pisa, Italy; 24 Department of Neurosciences, Psychiatry and Anaesthesiology, University of Messina, Messina, Italy

**Keywords:** Laminopathies, Emery-Dreifuss Muscular Dystrophy, Dilated Cardiomyopathy with Conduction Defects, Mandibuloacral Dysplasia, Familial Partial Lipodystrophy Type 2, Hutchinson-Gilford Progeria Syndrome, Rare Diseases, Networking activity, interdisciplinary approach to diseases

## Abstract

The need for a collaborative approach to complex inherited diseases collectively referred to as laminopathies, encouraged Italian researchers, geneticists, physicians and patients to join in the Italian Network for Laminopathies, in 2009. Here, we highlight the advantages and added value of such a multidisciplinary effort to understand pathogenesis, clinical aspects and try to find a cure for Emery-Dreifuss muscular dystrophy, Mandibuloacral dysplasia, Hutchinson-Gilford Progeria and forms of lamin-linked cardiomyopathy, neuropathy and lipodystrophy.

## Correspondence

Empowerment is the process of increasing the capacity of individuals or groups to make choices and to transform those choices into desired actions and outcomes. Empowerment is a necessity for patients with rare diseases, which are chronic, difficult to manage, so rare that coordinated efforts are imperative to make progress, and largely disregarded by the research or medical community and policy makers.

Individual empowerment is a reality for patients with rare diseases, which has been mediated by the rapid growth of web-based health-related information. Thus, a patients need of knowledge and support leads to the need for associations, which are able to collect and analyze the data of individual cases and present the lessons learned from each case in the context of the disease as a whole. The need for networking comes from the above-mentioned situation, yet, in the case of laminopathies, the need comes also from the nature and complexity of diseases.

The story of laminopathies began with the discovery, in 1994, that the Emery-Dreifuss muscular dystrophy was caused by mutations in *EMD* gene encoding a protein of the nuclear envelope [1]. During the following ten years, more than twelve diseases have been linked to mutations in lamin A/C [2], a main component of the nuclear lamina. Along with the lamin proteins other nuclear envelope proteins have also been associated with rare inherited disorders. All these diseases are currently refereed to as laminopathies. They include Emery-Dreifuss muscular dystrophy, limb-girdle muscular dystrophy type 1B, dilated cardiomyopathy with conduction defects, Dunnigan type familial partial lipodystrophy, Mandibuloacral dysplasia [3] and Hutchinson-Gilford progeria syndrome, atypical-Werner syndrome, Heart-hand syndrome, Charcot Marie-Tooth neuropathy type 2B1, restrictive dermopathy, autosomal dominant leucodystrophy, osteopoikilosis and other partially overlapping diseases [4].

Each laminopathy presents with typical clinical features, yet several aspects are shared by muscle and adipose tissue laminopathies or by premature ageing diseases and lipodystrophies. Thus, an interdisciplinary clinical approach is expected to yield better diagnosis, better follow-up and therapeutic chances. In fact, the most relevant advances in understanding the pathogenesis of lamin-linked diseases and of lamin function have been obtained after the discovery of syndromic laminopathies. The study of syndromic laminopathies, based on their molecular, cellular and clinical aspects, suggested major pathogenetic pathways to be explored in tissue-specific laminopathies. Moreover, given that most laminopathies affect tissues of mesenchimal origin and likely involve altered mesenchimal stem cell commitment or differentiation, pathogenetic mechanisms likely overlap and require a comprehensive view in order to unravel the role of lamin. These considerations provide evidence of the extent to which an Italian Network for Laminopathies, involving centers spread throughout Italy and involved in clinics, research, industry and patients and their associations can help in addressing the study of those diseases and finding a therapeutic strategy. As in any rare disease, the information is not well known among family doctors and even in specialized centers. In this context, a major aim of the Network is to expand knowledge and increase interest in diagnostic protocols and detection of symptoms.

Two main features of the Network make it efficient in reaching the objectives of an interdisciplinary approach to diagnosis, therapy and research activity. First, meetings of all Network partners are regularly held twice a year and researchers, clinicians and patients participate with data presentations, sharing of clinical aspects and suggesting new initiatives. Secondly, the Network website (http://www.igm.cnr.it/laminopatie/) is a platform to share scientific news, information about the diseases, available treatments and the location of knowledgeable clinicians. Successful completion of this information is the result of collaborations involving patients, physicians, other health-care providers and researchers.

A major result of the Network’s activity was the first Italian meeting course on Laminopathies, held in Bologna on April 2011 [5]. The meeting gave patients, family doctors and specialists in several disciplines the opportunity to be brought up-to-date on the most recent advances in the field of laminopathy diagnosis, clinical follow-up and research.

Finally, Network partners are developing collaborations in scientific and clinical projects. A critical mass of professionals, patients and biological samples gives unexpected opportunities to design comprehensive studies and search for biomarkers of disease, drug targets and pharmacological tools.

We propose that similar initiatives may be extended to other countries, involving people involved to different degrees in various aspects of laminopathies, to bring a multidisciplinary contribution to the understanding of diseases. Such an approach might prove useful for similar situations, especially when diverse clinical entities are associated with overlapping genetic and clinical tracts.

## Competing interests

The authors declare that they have no competing interests.

## Authors’ contribution

All the listed authors: 1) have made substantial contributions to conception and design of this letter; 2) have been involved in drafting the manuscript or revising it critically for important intellectual content; 3) have given final approval of the version to be published.

## Authors’ information

Enrico Bertini, Nicola Carboni, Adele D’Amico, Rocco Liguori, Eugenio Mercuri, Tiziana Mongini, Lucia O. Morandi, Giovanni Nigro, Antonella Pini, Stefano Previtali, Giulia Ricci, Carmelo Rodolico, Gabriele Siciliano are involved in Emery-Dreifuss Muscular Dystrophy clinical management. Elena Biagini, Giuseppe Boriani, Marianna Fontana, Luisa Politano, Claudio Rapezzi are involved in Dilated Cardiomyopathy clinical management. Monica D'Adamo, Alessandra Gambineri, Laura Mazzanti, Iria Neri, Renato Pasquali, Paolo Sbraccia, Emanuela Scarano are involved in Hutchinson-Gilford Progeria, Familial Partial Lipodystrophy type 2 and Mandibuloacaral Dysplasia clinical management. Sara Benedetti, Pia Bernasconi, Cristina Capanni, Giovanna Cenacchi, Marta Columbaro, Maria Rosaria D’Apice, Giovanna Lattanzi, Nadir M. Maraldi, Giuseppe Novelli, Michela Ortolani, Stefania Petrini, Lisa Pucci, Stefano Squarzoni are involved in basic, translational and clinical research on laminopathies.
